# Microstructure of the Working Layer of X46Cr13 Steel in a Bimetal System with Gray Cast Iron

**DOI:** 10.3390/ma17235933

**Published:** 2024-12-04

**Authors:** Natalia Przyszlak, Tomasz Wróbel, Agnieszka Dulska, Paweł M. Nuckowski, Dariusz Łukowiec, Marcin Stawarz

**Affiliations:** 1 Department of Foundry Engineering, Silesian University of Technology, 7 Towarowa Street, 44-100 Gliwice, Poland; tomasz.wrobel@polsl.pl (T.W.); agnieszka.dulska@polsl.pl (A.D.); marcin.stawarz@polsl.pl (M.S.); 2 Materials Research Laboratory, Faculty of Mechanical Engineering, Silesian University of Technology, 18A Konarskiego Street, 44-100 Gliwice, Poland; pawel.nuckowski@polsl.pl (P.M.N.); dariusz.lukowiec@polsl.pl (D.Ł.)

**Keywords:** high-chromium steel, gray cast iron, heat treatment, layered castings

## Abstract

The research conducted in this study aimed to determine whether the production of a layered casting in the material system of X46Cr13 steel (working part) and gray cast iron (base part) can be integrated with the hardening process of this steel within the conditions of the casting mold. Accordingly, a series of layered castings was produced by preparing the mold cavity, where a monolithic steel insert was poured with molten gray cast iron with flake graphite. The variable factors in the casting production process included the pouring temperature T_p_ and the thickness of the support part g. Importantly, given that the hardening of the X46Cr13 steel insert occurred directly within the mold, the selection of casting parameters had to balance the ability to heat the insert to the austenitization temperature Tγ_≥950°C_ while also creating thermokinetic conditions conducive to the rapid cooling of the system. Therefore, chromite sand—commonly regarded as a rapid-cooling material—was selected as the matrix for the molding material. Based on the conducted studies, it was determined that the thermokinetic properties of this material allowed the surface of the cast working part to be heated to the austenitization temperature. The microstructure consisted of Cr(Fe) carbides within a martensitic-pearlitic matrix, with martensite filling the grains of the primary austenite and pearlite situated along their boundaries. The carbides were primarily located at grain boundaries and, to a lesser extent, within the primary austenite grains. Through transmission electron microscopy and X-ray diffractometry, the type of Cr(Fe) carbide in the microstructure of the working part was identified as M_23_C_6_.

## 1. Introduction

Gray cast iron is the most widely used material for casting worldwide. This is due to the relatively low production costs of this material, its good technological properties, and other advantages such as its vibration damping and high thermal conductivity. In general, the properties of gray cast iron with flake graphite can be considered average. It is commonly used for components where high thermal conductivity and vibration damping are essential, such as engine housings, cylinders, engine blocks, and other machine parts [1,2,3].

Ductile cast iron also belongs to the gray iron group, but due to its unique spherical graphite structure and the resulting benefits, it is treated as a separate type of cast iron. Ductile cast iron has a higher tensile strength, impact resistance, and elastic modulus than gray cast iron with flake graphite. When subjected to heat treatment, it allows for further improvement in its mechanical properties. However, the production process of spheroidal cast iron is more complex and costly than that of gray cast iron. It requires precise control of the graphite modification process and the use of alloying additives such as magnesium or cerium, which increases the costs of materials and energy [4,5,6,7,8].

The dynamic development of many industries creates a continuous need for actions focused mainly on three aspects: simplifying processes, saving time, and minimizing costs. These demands also apply to the machinery industry, which increasingly requires castings with special properties, such as high resistance to abrasive wear and corrosion, and being made from expensive and hard-to-obtain materials [9,10]. Often, however, high-performance requirements apply only to the casting surfaces that are directly exposed to destructive external conditions (working surfaces) and consequently subject to wear. Therefore, producing such castings entirely from high-performance materials seems financially unjustifiable. As a result, layered casting technology (cast bimetals) becomes particularly important, which is applicable when only the working surface layer of the casting is used, while the rest serves merely as a supporting structure, not exposed to factors causing, for instance, corrosive or abrasive wear [11,12,13,14,15,16]. Although layered casting technology seems to be a very attractive production method, bimetals still hold a secondary status among structural and functional materials, possibly because practical applications lag behind experimental and theoretical research in this field [17,18,19,20].

Alloy steels, including corrosion-resistant steels, are materials with high amounts of mechanical and anti-corrosive properties, and numerous scientific publications indicate that cast bimetals with such steels as the working component and flake graphite gray cast iron as the structural component have been effectively produced [11,12,13,16,21,22]. For layered castings in this material configuration, it is worth optimizing the production process so that the working layer (made of steel) attains the highest possible operational properties. Ideally, selecting a steel grade with a high hardenability and appropriately choosing casting parameters for gray cast iron would make it possible to carry out heat treatment operations, such as steel quenching, within the casting mold conditions. This would integrate with the layered casting production process, thus enhancing its mechanical properties. Such a procedure aligns with modern manufacturing market expectations, focused on saving time and costs. While steel quenching performed under conventional conditions is a well-known and relatively easy operation, performing it directly within the casting mold appears to be more complex. Martensitic transformation requires heating the steel to its austenitizing temperature Tγ and rapid cooling. If this treatment is carried out in the casting mold, casting parameters should balance the heating of the steel to the Tγ and create thermokinetic conditions that are favorable for the rapid cooling of the system. However, there are no scientific publications on this subject, presenting an opportunity for research in this area [23,24,25].

Accordingly, the aim of this research was to attempt to combine the production of a layered casting in the high-chromium steel–gray cast iron system with the steel hardening process. For the integration of steel heat treatment with the layered casting production process, it is necessary to select a molding sand with a heat conduction coefficient high enough to allow for rapid heat dissipation from the casting through the mold to the environment. This approach would enable the cooling of the steel insert (the working part of the layered casting) at a rate required to achieve a martensitic structure. The thermophysical properties of the molding sand, affecting the rate of heat transfer, depend on the type of matrix used, as well as the size, shape, and packing of its grains [26,27]. Quartz sand, a commonly used and inexpensive molding material, has insufficient thermal properties. Therefore, in the experiment, chromite sand, known for its rapid cooling properties, was selected as the molding sand matrix [28,29,30,31,32,33].

## 2. Materials and Methods

**Figure 1:**
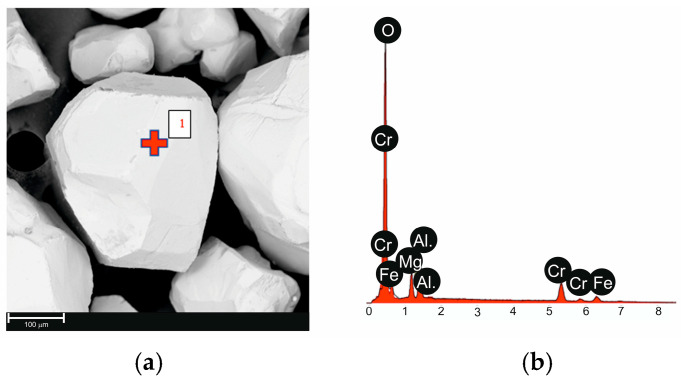
Chromite sand grain with marked location-of-point EDS analysis (**a**), EDS analysis result at point 1 (**b**).

The layered castings were produced by preparing the mold cavity with a monolithic X46Cr13 steel insert. The steel insert, measuring 50 × 50 × 5 mm, served as the working part of the bimetal. Gray cast iron grade EN-GJL-250 was used for the base part. The castings were made in a mold of chromite sand with grain sizes of 0.40 mm/0.32 mm/0.20 mm (coarse sand). The binder used was Carbophen 9026 resin, hardened with CO_2_. Figure 1 and Table 1 present the results of EDS analyses of the matrix grains, which confirm their chemical composition as expected.

The chemical compositions of the components of the layered casting are presented Table 2 and Table 3.

The experimental plan included the production of model layered castings within defined levels of variability for selected casting process factors and recording the temperature at the thermal center on the outer surface of the steel insert.

**Figure 2:**
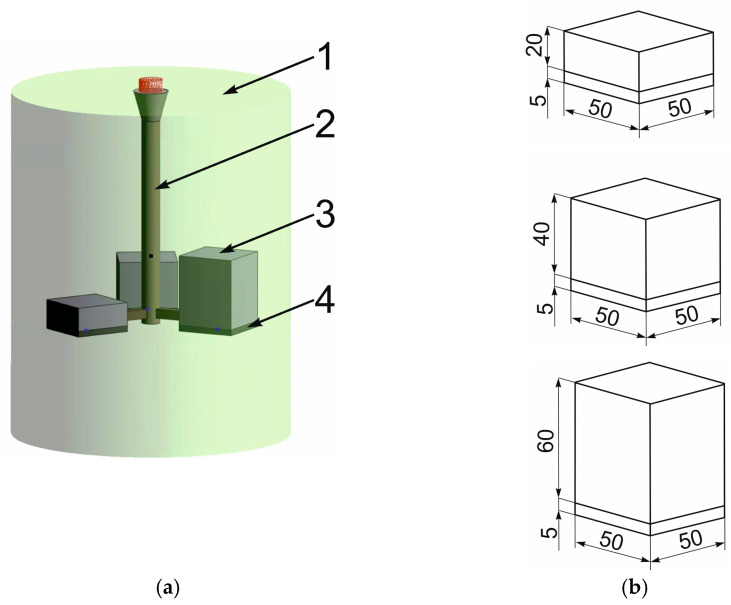
Sketch and dimensions [mm] of (**a**) the casting mold: 1−mold, 2−gating system, 3−base part of layered casting, 4−working part of the layered casting; (**b**) model layered castings.

The variable factors in the process were the thickness of the cast iron base part g and its pouring temperature T_p_. Both factors were analyzed at two levels of variability. The pouring temperatures were set at 1400 °C, 1450 °C, and 1500 °C. For each pouring temperature, castings were made with varying thicknesses of the cast iron section: 20, 40, and 60 mm (Figure 2). The experimental plan, covering the production of 9 layered castings, is presented in Table 4.

The insert was sandblasted and coated with a flux–water solution of Na_2_B_4_O_7_ and H_3_BO_3_, then dried at 120 °C for 1 h to remove H_2_O, before being placed in the mold cavity. To simplify the technology, it was decided to not preheat the insert, which, as demonstrated in studies [14,24,34,35] for a similar material configuration, does not preclude achieving a durable connection between the two alloys.

Summarizing, the manufacturing process involved the following:

1. The working part of the layered casting was made of high-chromium steel X46Cr13 steel grade, whereas for the base part, gray cast iron with the flake graphite was used.
2. The casting was manufactured using molding mass based on chromium sand, and the bonding agent was Carbophen 9026 resin, hardened with CO_2_.
3. The insert, before being placed in the mold cavity and poured by the molten gray cast iron, was sandblasted and coated with a flux based on the boron and sodium compounds to remove barriers to achieving a durable connection between the steel (working part) and the gray cast iron (base part).
4. The casting produced in the range of studies differed in the thickness of the base part (amounted to 20, 40, or 60 mm), as well as in pouring temperature (amounted to 1400, 1450, and 1500 °C).

During the pouring and cooling of the casting, the temperature at the thermal center of the outer surface of the steel insert was recorded to determine the maximum temperature T_m_ that the outer surface of the working layer of the casting reached. Additionally, based on the recorded curves T = F(t), the cooling rate V_8/5_ on the outer surface of the working part of the casting was determined, i.e., the cooling rate in the temperature range of 800–500 °C.

**Figure 3:**
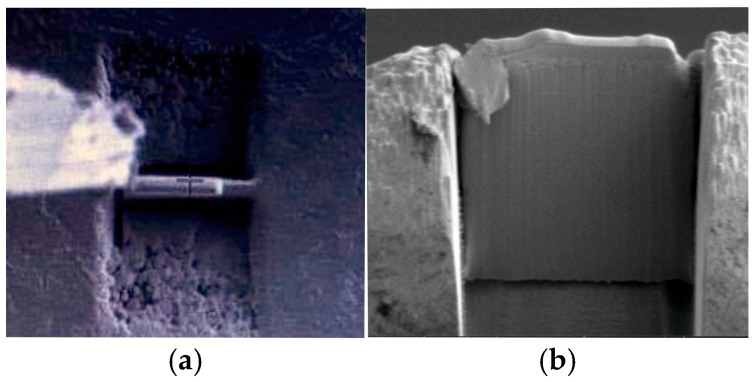
Method of obtaining lamellae for TEM studies: (**a**) top view, mag. 4500×, (**b**) side view, mag. 6500×.

The efficiency of the bond between the working layer and the base part of the bimetal, i.e., between the high-chromium tool steel and the gray cast iron, was investigated on a Starmans ElektronicsDIO 1000 defectoscope (STARMANS ELECTRONICS S.R.O., Prague, Czech Republic). Microstructure studies and phase composition analysis in the layered castings, with particular focus on the working layer, were conducted using scanning electron microscopy (SEM), and transmission electron microscopy (TEM). A qualitative X-ray diffraction analysis (XRD) was also performed. Metallographic studies were conducted using microscopy, involving grinding with sandpapers of grits 200 to 1000, polishing on a felt disk with an aqueous Al_2_O_3_ solution, and electrolytic etching in Mi19Fe reagent composed of 3 g of FeCl_3_, 10 cm^3^ of HCl, and 90 cm^3^ of C_2_H_5_OH, at a voltage of 15 V and an etching time of 20 s. Microstructure observations were carried out using a Phenom ProX scanning electron microscope (SEM) INSPECT F (FEI Technologies Inc., Hillsboro, OR, USA) equipped with an energy-dispersive X-ray spectroscopy (EDS) spectrometer. In the SEM studies, imaging was performed in the backscattered electron (BSE) mode at accelerating voltages of 10 and 15 Kv, employing point, linear, and surface microanalysis of the chemical composition using the EDS method. Studies using the transmission electron microscope were performed using the thin foil method. Thin foils (lamellae) with a thickness of 50 nm were obtained using the microscope and a gallium ion beam (SEM/Ga-FIB) FEI Helios NanoLab 600i. A representative method for obtaining the lamellae is illustrated in Figure 3.

Microstructure observations of the thin foils were conducted using a S/TEM TITAN 80–300 transmission electron microscope from FEI (FEI Technologies Inc., Hillsboro, OR, USA), operating at an accelerating voltage of U = 300 kV and a wavelength of λ = 0.00197 nm. Identification of the observed phases was performed using the electron diffraction method. The analysis of the diffraction pattern involved measuring the lengths of five selected reflections from the central point of the undiffracted beam, as well as the angles between the rays of these reflections. In the next step, the interplanar spacings specific to each reflection were determined based on the relationship (1):

(1) $$d=\frac{\lambda L}{r},$$

where *L* is the interplanar spacing, *λ* is the wavelength of the electron beam, *r* is the distance of the reflection from the zero point.

The calculated interplanar spacings were then compared with tabulated values, and the appropriate indices for the plane families {hkl} were assigned. Subsequently, specific indices for the planes {hkl} that satisfy the vector relationship were established (2):

(2) $${\bar{r}}_{1}\;+\;{\bar{r}}_{3}={\bar{r}}_{2}\;i\;{\bar{r}}_{3}\;+\;{\bar{r}}_{5}={\bar{r}}_{4}$$

corresponding to the dependencies: [h_1_k_1_l_1_] + [h_3_k_3_l_3_] = [h_2_k_2_l_2_] and [h_3_k_3_l_3_] + [h_5_k_5_l_5_] = [h_4_k_4_l_4_].

Finally, the measured values of the interplanar angles were compared with tabulated data for the specific phase, and the indices of the crystallographic zone axis [uvw] of the studied phase were determined from Equations (3)–(5), where h_1_k_1_l_1_ and h_2_k_2_l_2_ are the Miller indices of two selected reflections on the diffractogram belonging to the same crystallographic zone [36].

u = k_1_l_2_ − l_1_k_2_ (3)

v = l_1_h_2_ − h_1_l_2_ (4)

v = l_1_h_2_ − h_1_l_2_ (5)

X-ray phase analysis was conducted using the X’Pert Pro device from Panalytical (Panalytical B.V. (currently: Malvern Panalytical Ltd.), Almelo, The Netherlands), operating at a voltage of 40 kV and a current of 30 mA. Measurements were performed in the angular range of 2θ from 10° to 110° with a recording step of 0.05°. X-ray investigations were carried out using radiation from a Co anode with an Fe filter in a configuration with a Pixcel detector. Phase identification was performed according to data from the International Centre for Diffraction Data (ICDD).

## 3. Results and Discussion

All the castings exhibited a high degree of bonding between the supporting and working parts [37]. The set of thermal and kinetic parameters measured and calculated according data collected with thermoelemnts are presented in Table 3: Tm—temperature to which the outer surface of steel insert was heated; t_800_ and t_500_—time required to heat the steel insert to the indicated temperature; t_>950_—dwell time above the austeinitization temperature; V_8/5_—cooling rate. The austenitization temperature range for steel X46Cr13 is 950 to 1050 °C [38]. The use of a mold material based on chromite sand allowed for surpassing the lower limit of the austenitization temperature for all castings. The temperature defining the upper limit of austenitization was achieved by the castings where the thickness g of the base part was the largest at 60 mm, regardless of the pouring temperature T_p_ (C3, C6, C9) and for casting C8, where T_p_ was 1500 °C and g = 40. The requirements for the austenitization time were also met for the aforementioned castings.

According to the data in the literature [36], the proper austenitization time, i.e., the duration for which the material remains at the austenitization temperature, for a plate with a thickness of 5 mm is 450 s. This goal was achieved only for the castings where the height of the base part was the greatest at 60 mm, regardless of the pouring temperature used. It should be noted that only for these castings (i.e., C3, C6, and C9) was the steel insert hardened throughout its volume (Table 5).

Based on temperature measurements as a function of time during the pouring and cooling of the casting, the cooling rate of the steel insert in the temperature range of 800 to 500 °C was also determined. According to the analysis of the cooling rate curves for the selected steel grade, rapid cooling in the temperature range of 800 to 500 °C is crucial for inducing the martensitic transformation in the steel X46Cr13 used for the insert. It should also be noted that phase transformations typical for the hardening process pertain only to the matrix of chromium (iron) carbides, which precipitate in X46Cr13 steel regardless of its cooling parameters from the austenitization temperature. An increase in the cooling rate V_8/5_, as assumed, should result in an increase in the amount of martensite, at the expense of reducing the amount of other phases, e.g., pearlite. It was found that at a given pouring temperature, the greatest cooling rate of the insert was achieved by using the lowest height of the base part (20 mm).

**Figure 4:**
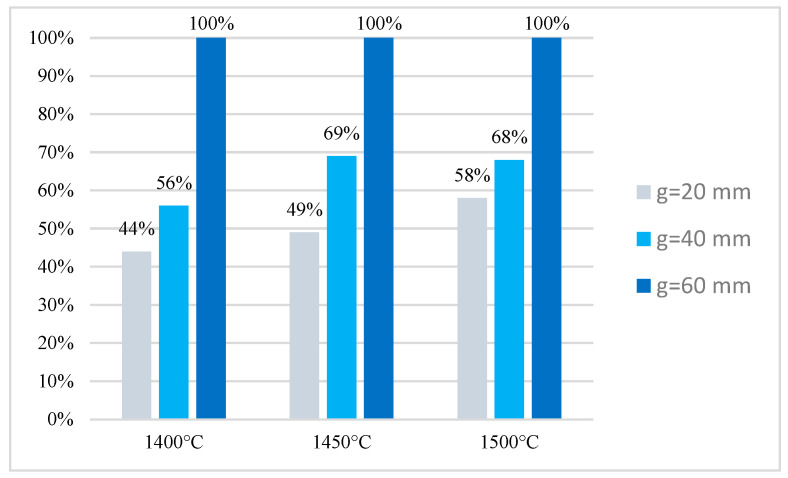
Results of defectoscope examinations: The size of the surface P on which the base part is permanently connected with the working part in the tested layered castings.

**Figure 5:**
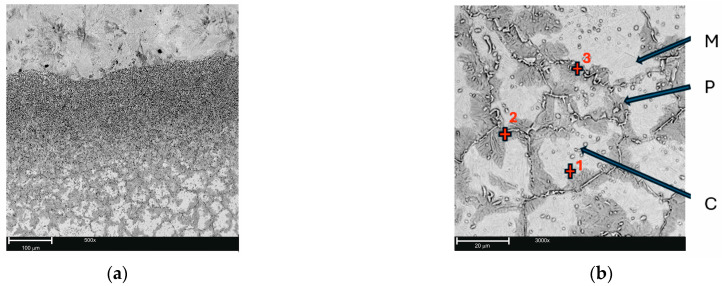

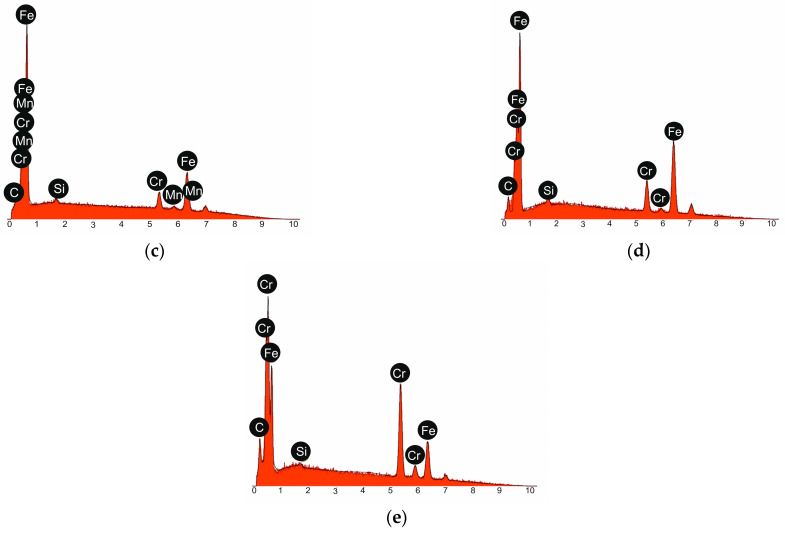
(**a**) Example microstructure of layered casting: gray cast iron EN-GJL-HB 255—steel X46Cr 13 (casting no. C3) SEM, mag. 500×. (**b**) Microstructure of X46Cr 13 steel containing Cr(Fe)—(C) carbides in a martensitic–pearlitic matrix (M-P) in the working layer of the bimetal (casting no. C8) with marked locations for point EDS analysis, SEM, mag. 6000×. (**c**) EDS analysis result at point 1. (**d**) EDS analysis result at point 2. (**e**) EDS analysis result at point 3.

Based on the results of metallographic microscopic studies, it was determined that in the areas of layered castings, where defectograms were identified as a proper connection between X46Cr13 steel (insert) and gray cast iron (casting alloy) (Figure 4), diffusion of components occurred, leading to the formation of so-called transition zones (Figure 5a). The gradient character of the microstructure in the area of the joint was due to the diffusive transport of components, primarily carbon, towards a lower concentration, i.e., towards the steel insert. Consequently, the base part zone consisted of flake graphite in a pearlitic matrix. As one moved from the base part layer towards the steel insert, a zone with a pearlitic microstructure, a zone with Cr(Fe) carbides in a pearlitic matrix, and a zone with Cr(Fe) carbides in a pearlitic matrix containing a small amount of martensite were identified. From the perspective of the tribological properties of the layered casting, the microstructure of the working layer of this casting was crucial. It consisted of Cr(Fe) carbides in a martensitic–pearlitic matrix (Figure 5b–e and Table 6).

**Figure 6:**
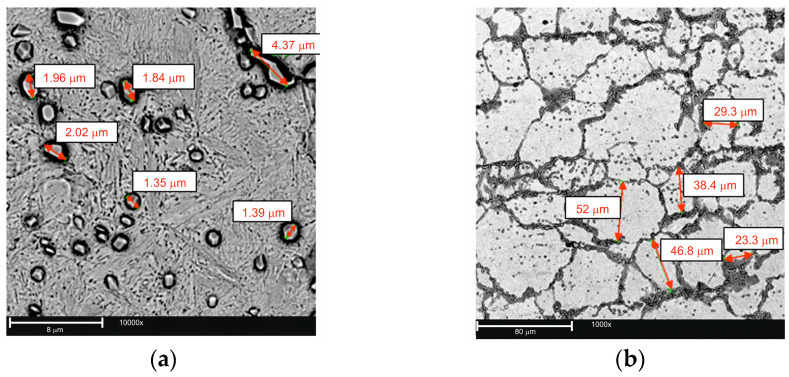
Example microstructures of the working layer in the bimetal gray cast iron EN-GJL-HB 255–steel X46Cr 13, SEM: (**a**) Cr(Fe) carbides in a and martensitic matrix (casting no. C2), mag. 10,000×. (**b**) Size of a pearlitic martensitic matrix grain (casting no. C8), mag. 3000×.

The average size of Cr(Fe) carbides was approximately 2 µm (Figure 6a). The carbides were mainly located along the boundaries and to a lesser extent within the grains of the primary austenite, which had an average size of about 30 µm (Figure 6b).

**Figure 7:**
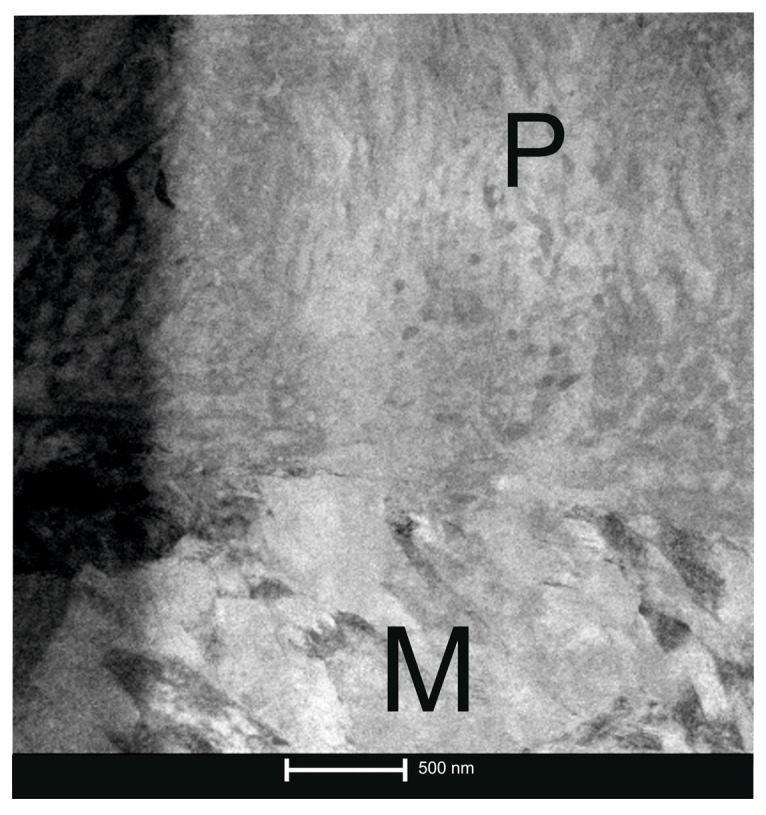
Phase boundary between pearlite—P—(above) and martensite—M—(below) in the microstructure of the working part of the layered casting gray cast iron EN-GJL-HB 255–steel X46Cr 13, TEM, mag. 15,000×.

**Figure 8:**
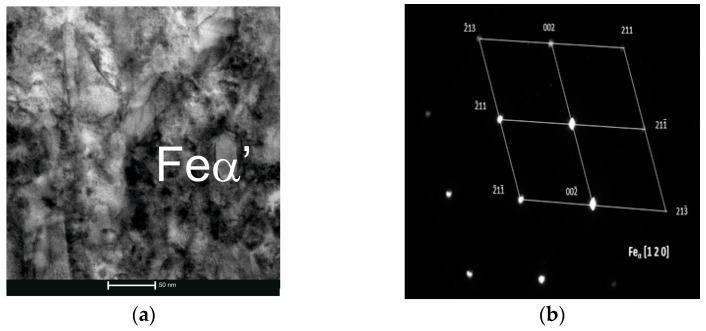
Feα in the microstructure of the working part of the layered casting gray cast iron EN-GJL-HB 255–steel X46Cr 13: (**a**) bright field TEM, mag. 60,000×; (**b**) SAD diffraction.

**Figure 9:**
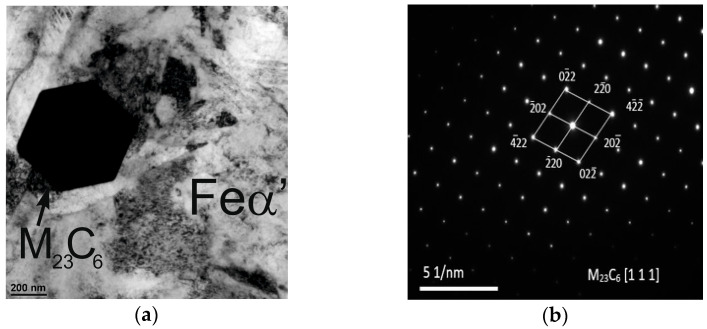
M_23_C_6_ carbide in the microstructure of the working part of the layered casting gray cast iron EN-GJL-HB 255–steel X46Cr 13: (**a**) bright field, TEM, mag. 30,000×; (**b**) SAD diffraction.

Based on the results obtained using transmission electron microscopy and X-ray diffraction, the type of Cr(Fe) carbide in the microstructure of the working layer of the bimetal was identified as M_23_C_6_ (Figure 7, Figure 8 and Figure 9 and Table 7), which is supported by the findings presented in references [39,40]. Furthermore, it was shown that the parameters T_p_ and g do not influence the presence of M_23_C_6_ carbides in the microstructure of X46Cr13 steel. In addition to peaks originating from the Feα and M_23_C_6_ phases, reflections from cementite M_3_C were identified in some castings, which in these cases should be considered a component of pearlite (Table 7). Based on the obtained results, it was concluded that, essentially, regardless of the parameters used for casting the bimetal, the microstructure of the working layer qualitatively consists of Cr(Fe) carbides in a martensitic–pearlitic matrix, with martensite filling the grains of primary austenite and pearlite located at their boundaries.

## 4. Conclusions

The objective of the research was to integrate the production of a layered casting in the system of high-chromium steel X46Cr13–gray cast iron EN-GJL-250 with the hardening of the aforementioned steel. The castings were made in a mold based on chromite sand, which, according to the literature, has a higher heat conductivity coefficient than the commonly used quartz sand. To achieve the hardening of the 5 mm thick steel, it was necessary to heat it to the austenitization temperature, hold it at this temperature for 450 s, and then cool it at a rate greater than the critical rate. The first hardening condition was met for all castings—the external surface of the insert was heated to above 950 °C. The second condition, ensuring the full hardening of the steel throughout its volume, was met only for those castings where the thickness of the load-bearing part was the largest, measuring 60 mm. However, in the steel microstructure, alongside martensite and carbides, pearlite was also present. Referring to the research results presented in the paper, the key factor influencing the presence of pearlite in the working layer of X46Cr13 steel was the too low cooling rate of the system. In addition, based on the conducted analyses, the following conclusions were drawn:

- Microscopic examination of the working layer revealed a microstructure predominantly composed of martensite and pearlite, along with Cr(Fe) carbides.
- Based on studies using a transmission electron microscope and an X-ray diffractometer, the type of Cr(Fe) carbide in the microstructure of the working layer was identified as M_23_C_6_.
- The relatively high cooling rate of castings made with molding sand based on chromite sand resulted in a low pearlite content relative to martensite in the microstructure of the working layer, where pearlite was primarily located along the grain boundaries of martensite.
- Further research in the analyzed range should focus on optimizing the production process for layered castings made with FeCr_2_O_4_-based molding sand, aiming to minimize the pearlite in the microstructure of the working part. This reduction is expected to further enhance the hardness of the working surface made from X46Cr13 steel.

## Figures and Tables

**Table 1:** Result of EDS analysis for point 1 from 1.

Element	%at.	%wt.
O	60.14	35.41
Cr	16.00	30.63
Fe	10.16	20.88
Al	8.31	8.26
Mg	5.39	4.82

**Table 2:** Chemical composition of X46Cr13 steel.

C	Cr	Ni	Mo	Mn	Si	Al	Cu	V	S	P
0.43	13.6	0.125	0.015	0.61	0.383	0.003	0.069	0.099	0.002	0.025

**Table 3:** Chemical composition of EN-GJL-HB 255.

C	Mn	Si	Cr	Ni	Cu	Al	V	P	S
3.55	0.45	2.150	0.123	0.058	0.071	0.004	0.048	0.260	0.100

**Table 4:** Experimental plan.

Symbol of Casting	Thickness of the Base Part (g), mm	Pouring Temperature (T_p_), °C
C1	20	1400
C2	40	
C3	60	
C4	20	1450
C5	40	
C6	60	
C7	20	1500
C8	40	
C9	60	

**Table 5:** Thermal and kinetic parameters of the working parts of layered castings.

	C1	C2	C3	C4	C5	C6	C7	C8	C9
T_m_, °C	999	1021	1080	995	1024	1087	1001	1059	1137
t_800,_ s	284	504	1029	381	633	932	367	709	915
t_500,_ s	1217	1922	4141	1550	3327	3767	1939	3314	3691
t_>950_, s	131	234	495	116	245	466	107	356	481
V_8/5_, °C/s	0.32	0.21	0.10	0.26	0.11	0.10	0.19	0.11	0.10

**Table 6:** Results of EDS analysis for point 1, 2, and 3 from Figure 5b.

Measurement Point	Element	%at.	%wt.
Point 1 from Figure 5b	Fe	81.9	83.8
	Cr	14.5	13.8
	Mn	1.7	1.7
	Si	0.8	0.4
	C	1.2	0.3
Point 2 from Figure 5b	Fe	73.4	82.6
	Cr	13.4	14.0
	C	12.5	3.0
	Si	0.7	0.4
	Fe	73.4	82.6
Point 3 from Figure 5b	Cr	43.1	48.2
	Fe	39.4	47.3
	C	17.4	4.5
	Si	0.1	0.1

**Table 7:** Exemplary X-ray diffractogram of the working part of the layered casting.

No of Casting	X-Ray Diffractogram of the Working Part of the Layered Casting
C3	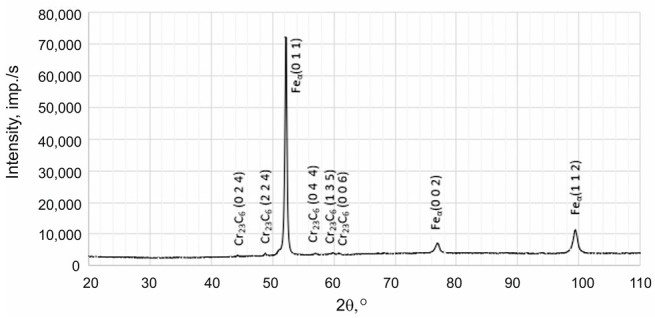
C6	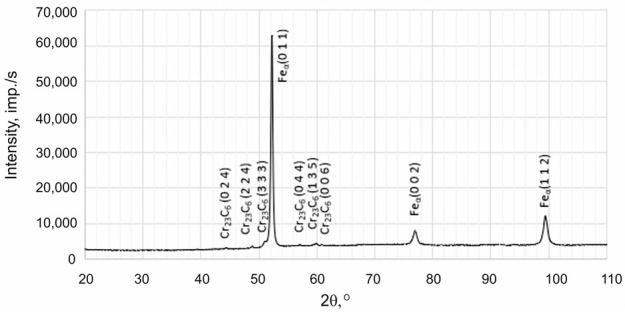
C9	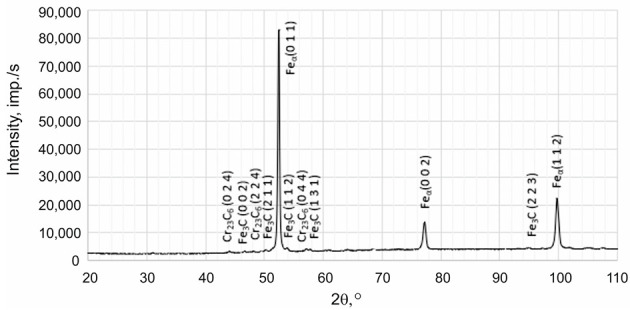

## Data Availability

Data are contained within the article.

## References

[1] Dwulat R., Janerka K., Grzesiak K., Gałuszka M. (2023). Influence of Charge Materials on the Metallurgical Quality of Gray Cast Iron. Arch. Foundry Eng..

[2] Mnati A.A., Resan K.K. (2023). Production of Ductile Cast Iron by Recycling Gray Cast Iron Scrap With Adding Various Local Materials. J. Sustain. Dev..

[3] Salawu E.Y., Elvis A.O., Ajayi O.O., Ongbali S.O., Afolalu S.A. (2023). Particle size distribution analysis of carburized HT250 gray cast iron using ImageJ. Mater. Today.

[4] Li Q., Zhang Y., Zhang Y., Liu H., Ren H., Zhong Y., Huang X., Huang W. (2020). Influence of Sn and Nb additions on the microstructure and wear characteristics of a gray cast iron. Appl. Phys. A.

[5] Masafi M., Palkowski H., Mozaffari-Jovein H. (2023). Microstructural properties of particle-reinforced multilayer systems of 316L and 430L alloys on Gray Cast Iron. Coatings.

[6] Aranke O., Algenaid W. (2019). Coatings for automotive gray cast iron brake discs. Coatings.

[7] Akinribide O.J., Ogundare O.D., Oluwafemi O.M., Ebisike K., Nageri A.K., Akinwamide S.O., Gamaoun F., Olubambi P.A. (2022). A Review on Heat Treatment of Cast Iron: Phase Evolution and Mechanical Characterization. Materials.

[8] Du S., Chen C., Chen R., Wang Q., Cui X., Song Q. (2024). Influence of Casting Materials on the Microstructure and Mechanical Properties of Gray Cast Iron for Cylinder Liners. Int. J. Met..

[9] Studnicki A., Kilarski J., Przybył M., Suchoń J. (2005). Technological experiments of production castings of chromium cast iron in the foundry. Arch. Foundry Eng..

[10] Bykov A. (2011). Bimetal Production and Applications. Steel Transl..

[11] Ramadan M. (2015). Interface characterization of Bimetallic Casting with 304 Stainless Steel Surface Layer and Gray Cast Iron Base. Adv. Mater. Res..

[12] Li Y., Gong M., Wang K., Li P., Yang X., Tong W. (2018). Diffusion behavior and mechanical properties of high chromium cast iron/low carbon steel bimetal. Mater. Sci. Eng. A.

[13] Ibrahim M.M., El-Hadad S., Mourad M. (2020). Effect of liquid-solid volume ratios on the interfacial microstructure and mechanical properties of high chromium cast iron and low carbon steel bimetal. Mater. Res. Express.

[14] Przyszlak N., Dulska A., Wróbel T., Szajnar J. (2018). Grey cast iron locally reinforced using 3D printing scaffold insert. Arch. Foundry Eng..

[15] Przyszlak N., Dulska A., Wróbel T. Local reinforcement titanium carbide TIC type manufactured in method of mould cavity preparation using 3D printing. Proceedings of the Annual Conference Metal.

[16] Wróbel T. (2024). Compound Castings for the Coke Industry. Materials.

[17] Zhang Y., Wang A., Liang T., Zhang J., Mao Z., Ji J., Xie J., Chang Q., Su B., Liu S. (2024). Texture characteristics and tensile deformation behavior of Cu/Al layered composites prepared by cast-rolling method. J. Mater. Res. Technol..

[18] Yesuvadian R.A., Keerthiprasad K.S. (2023). A Review on Casting and Testing of Bimetals. Int. J. Appl. Eng. Res..

[19] Wang Y., Qin F., Qi H., Qi H., Meng Z. (2022). Interfacial Bonding Behavior and Mechanical Properties of a Bimetallic Ring Blank Subjected to Centrifugal Casting Process. J. Mater. Eng. Perform.

[20] Greß T., Mittler T., Volk W. (2020). Casting methods for the production of rotationally symmetric copper bimetals. Mater. Sci. Technol..

[21] Xiong B., Cai C., Wan H., Lu B. (2011). Fabrication of high chromium cast iron and medium carbon bimetal by liquid-solid casting in electromagnetic induction field. Mater. Des..

[22] Xiong B., Cai C., Lu B. (2011). Effect of volume ration of liquid to solid on the interfacial microstructure and mechanical properties of high chromium cast iron and medium chromium carbon steel bimetal. J. Alloys Compd..

[23] Brytan Z. (2018). Vademecum of Stainless Steel.

[24] Wróbel T. (2014). Characterization of bimetallic castings with an austenitic working surface layer and an unalloyed cast steel base. J. Mater. Eng. Perform..

[25] Campbell J. (2015). Complete Casting Handbook—Metal Casting Processes, Metallurgy, Techniques and Design.

[26] Cholewa M., Baron C., Kozakiewiecz Ł. (2015). Effect of Thermal Insulating Molding Sand on the Microstructure of Gray Cast Iron. Arch. Foundry Eng..

[27] Jacquet P., Vaucheret A., Souêtre M., Carton J.F. (2024). Determination of Thermal Properties of Foundry Green Sand to Improve Numerical Simulation. Int. J. Met..

[28] Przyszlak N., Wróbel T. (2019). Self-hardening of X46Cr13 steel integrated with base from grey cast iron in bimetallic system. Arch. Foundry Eng..

[29] Przyszlak N., Piwowarski G. (2023). Designing of X46Cr13 Steel Heat Treatment in Condition of Casting Mould. Arch. Foundry Eng..

[30] Hess K., Ignaszak Z. Thermal Conductivity of Molding Sands as a Function of Temperature. Proceedings of the International Symposium—Solidification of Metals and Alloys.

[31] Li L., Lu Y., Ren T., Horton R. (2023). Quartz contents derived from particle density measurements improve the accuracy of soil thermal conductivity estimates. Geoderma.

[32] Zhang X.R., Kong G.Q., Wang L.H., Xu X.L. (2020). Measurement and prediction on thermal conductivity of fused quartz. Sci. Rep..

[33] He H., Li M., Dyck M., Si B., Wang J., Lv J. (2020). Modelling of soil solid thermal conductivity. Int. Commun. Mass Transf..

[34] Cholewa M., Wróbel T., Tenerowicz S., Szuter T. (2010). Diffusion phenomena between alloy steel and gray cast iron in layered bimetallic casting. Arch. Metall. Mater..

[35] Gawroński J., Szajnar J., Wróbel P. (2004). Study on theoretical bases of receiving composite alloy layers on surface of cast steel castings. J. Mater. Process. Technol..

[36] Marciniak J., Hajduczek E. (1978). Methods of Testing Metals and Alloys: Structural Metallography.

[37] Przyszlak N., Wróbel T., Dulska A. (2021). Influence of Molding Materials on the Self-Hardening of X46Cr13 Steel/Grey Cast Iron Bimetallic Castings. Arch. Metall. Mater..

[38] Staub F., Adamczyk J., Cieślakowa Ł., Gubała J., Maciejny A. (1994). Metallography.

[39] Garcia de Andrès C., Caruana G., Alvarez L. (1998). Control of M23C6 carbides in 0.45C–13Cr martensitic stainless steel by means of three representative heat treatment parameters. Mater. Sci. Eng..

[40] Yang Y., Zhao H., Dong H. (2020). Carbide evolution in high-carbon martensitic stainless cutlery steels during austenitizing. J. Mater. Eng. Perform..

